# Construction of Spindle-Shaped Ti^3+^ Self-Doped TiO_2_ Photocatalysts Using Triethanolamine-Aqueous as the Medium and Its Photoelectrochemical Properties

**DOI:** 10.3390/nano12132298

**Published:** 2022-07-04

**Authors:** Zunfu Hu, Qi Gong, Jiajia Wang, Xiuwen Zheng, Aihua Wang, Shanmin Gao

**Affiliations:** 1 School of Materials Science and Engineering, Linyi University, Linyi 276005, China; huzunfu@lyu.edu.cn (Z.H.); gongqi72909@163.com (Q.G.); wangjiajia@lyu.edu.cn (J.W.); 2 School of Chemistry & Chemical Engineering, Linyi University, Linyi 276005, China; zhengxiuwen@lyu.edu.cn; 3 Yantai North China Microwave Technology Co., Ltd., Yantai 264025, China; 13953598088@163.com

**Keywords:** titanium dioxide, Ti^3+^ self-doped, spindle-shaped, triethanolamine, photocatalytic

## Abstract

To enhance the utilization efficiency of visible light and reduce the recombination of photogenerated electrons and holes, spindle-shaped TiO_2_ photocatalysts with different Ti^3+^ concentrations were fabricated by a simple solvothermal strategy using low-cost, environmentally friendly TiH_2_ and H_2_O_2_ as raw materials and triethanolamine-aqueous as the medium. The photocatalytic activities of the obtained photocatalysts were investigated in the presence of visible light. X-ray diffraction (XRD), Raman spectra, transmission electron microscope (TEM), X-ray photoelectron spectroscopy (XPS), and Fourier transform infrared (FT-IR) spectra were applied to characterize the structure, morphologies, and chemical compositions of as-fabricated Ti^3+^ self-doped TiO_2_. The concentration of triethanolamine in the mixed solvent plays a significant role on the crystallinity, morphologies, and photocatalytic activities. The electron–hole separation efficiency was found to increase with the increase in the aspect ratio of as-fabricated Ti^3+^ self-doped TiO_2_, which was proved by transient photocurrent response and electrochemical impedance spectroscopy.

## 1. Introduction

As one of the most promising strategies to deal with the worldwide environmental and energy crises, photocatalytic (PC) technology takes particular attention of being a green technology that can be applied under solar conditions. PC activity is largely determined by the separation efficiency of photogenerated electrons and holes with high chemical energy [1,2]. Due to its earth abundance, nontoxic high-photoelectrical conversion performance [3], and strong chemical stability against photo and chemical corrosion, TiO_2_ has been extensively applied as photocatalyst for solar hydrogen production [4], photovoltaic power generation [5,6], sewage purification [7,8,9], air purification, and other fields [10,11]. Hence, TiO_2_-based photocatalysts with high PC activity have received continuous attention. However, two defects, wide band-gap (3.2 eV for pure TiO_2_) and rapid charge recombination rate, limit the large-scale application of TiO_2_-based photocatalysts [12,13]. Hence, PC activity may be enhanced by comprehensive studies on band engineering and charge carrier dynamics to solve the above problems of pure TiO_2_ photocatalyst [14,15].

Many strategies have been developed during the past few decades to enhance the absorption of visible light [1,16]. Heteroatom doping is the most basic way of changing the electronic structure of functional materials; for instance C [17], S [18,19], and N doping [20,21], acting as electronic donation, and receipt have long been actively used to modify wide band-gap TiO_2_ photocatalysts for visible light absorption. In addition, surface modification strategies also were applied to improve the PC activity of TiO_2_, such as transition metal doping, dye sensitization, inorganic combination, and nanostructure-tuning [22]. These efforts usually suffer from the leakage of harmful metal ions, thermal instability, and the increase in deleterious recombination centers for photogenerated charges [23,24,25,26]. These vital drawbacks have increased the limited use of TiO_2_-based materials in developing photocatalysts. Therefore, reasonable modification strategies play a major role in improving PC properties of TiO_2_-based photocatalysts.

As a typical n-type semiconductor, TiO_2_ possesses intrinsic oxygen deficiency. The introduction of Ti^3+^ and oxygen vacancies (OVs) was employed to broaden the visible light absorption range and promote the charge separation [13,27,28]. Hence, Ti^3+^ self-doped TiO_2_ nanomaterials have been widely investigated for their extended visible light absorption and high conductivity [29,30]. Inducting Ti^3+^ and OVs would create new defect states below the conduction band of TiO_2_, thus increasing its PC activity [31,32,33,34]_._ Thus far, various strategies for introducing Ti^3+^ are often used; examples are thermal annealing at a high temperature in various reducing atmospheres, such as H_2_, CO, H_2_/Ar, or vacuum [35,36,37]. Nevertheless, the above strategies face the problems of high equipment requirements and high manufacturing costs. Hence, developing a facile strategy to construct Ti^3+^ self-doped TiO_2_ is still a potential research topic.

Bulk and surface studies cannot elucidate the shape dependence effect, which is regarded as the origin of the higher performance of smaller particles. Serial organic surfactants, such as PVP [38], PEG [39], CTAB [40], thiophenol [41], and TEA [42] have been employed to tune the size and morphology and to prevent agglomeration. The introduction of surfactant could also stabilize the active adsorption sites that are conducive to the formation of smaller TiO_2_-based photocatalysts [43,44]. In our previous work, reduced TiO_2_ photocatalysts with different OVs contents were prepared by hydrothermal method, and the effects of hydrothermal treatment temperature and time consumed on the structure, morphology, and properties of the obtained samples were studied [45,46]. However, the solvent effect has an important impact on the morphology of the product, thus affecting the PC performance of the products. As a soluble, inexpensive, and readily available organic surfactant, triethanolamine (N(CH_2_CH_2_OH)_3_, TEA) plays a vital role in the regulation of crystallinity, morphology, and electronic structure of the TiO_2_-based photocatalysts [47,48,49,50,51,52,53]. Herein, to optimize the PC performance of TiO_2_, a facile mixed solvothermal method was developed to fabricate spindle-shaped Ti^3+^ self-doped TiO_2_ with TEA as structure-directing and capping agent.

## 2. Construction of Spindle-Shaped TiO_2_ Photocatalysts with Ti^3+^ and OVs

**Scheme 1:**
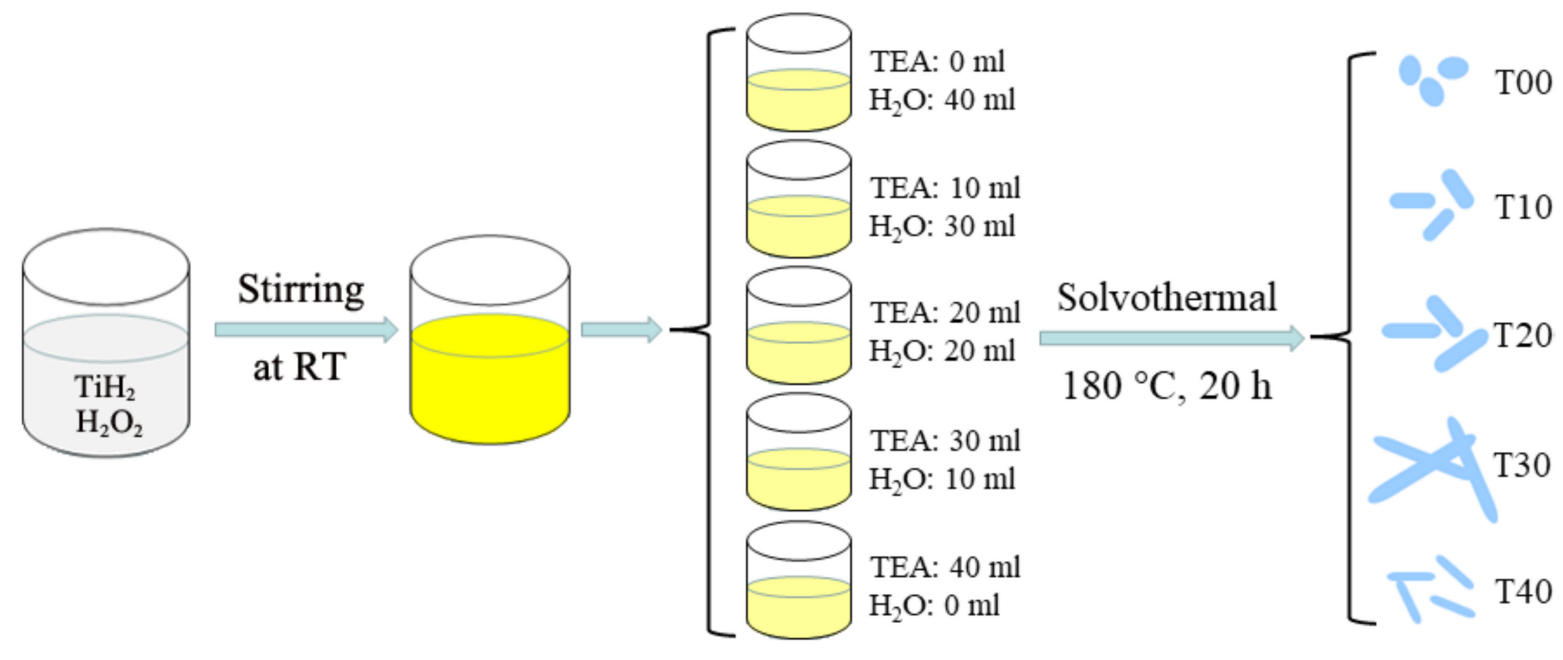
Schematic diagram of the synthesis route of Ti^3+^ self-doped TiO_2_ photocatalysts.

Ti^3+^ self-doped TiO_2_ photocatalysts were prepared with TiH_2_ as Ti source, H_2_O_2_ as oxidant, and TEA as structure-directing agent. The entire synthesis process is illustrated in Scheme 1. The sol obtained by oxidizing TiH_2_ with H_2_O_2_ was evenly divided into five parts, and then different amounts of TEA and H_2_O were added, respectively. The solvothermal reaction subsequently was conducted at 180 °C for 20 h. The products were cleaned with deionized water and absolute ethanol three times, and centrifugate was collected. After drying at 60 °C, light blue photocatalysts were obtained.

The chemicals, detailed preparation process, characterization, PC, and photoelectrochemical measurement experiment of the spindle-shaped TiO_2_ with Ti^3+^ self-doped are detailed in the Supplementary Materials.

## 3. Results and Discussion

**Figure 1:**
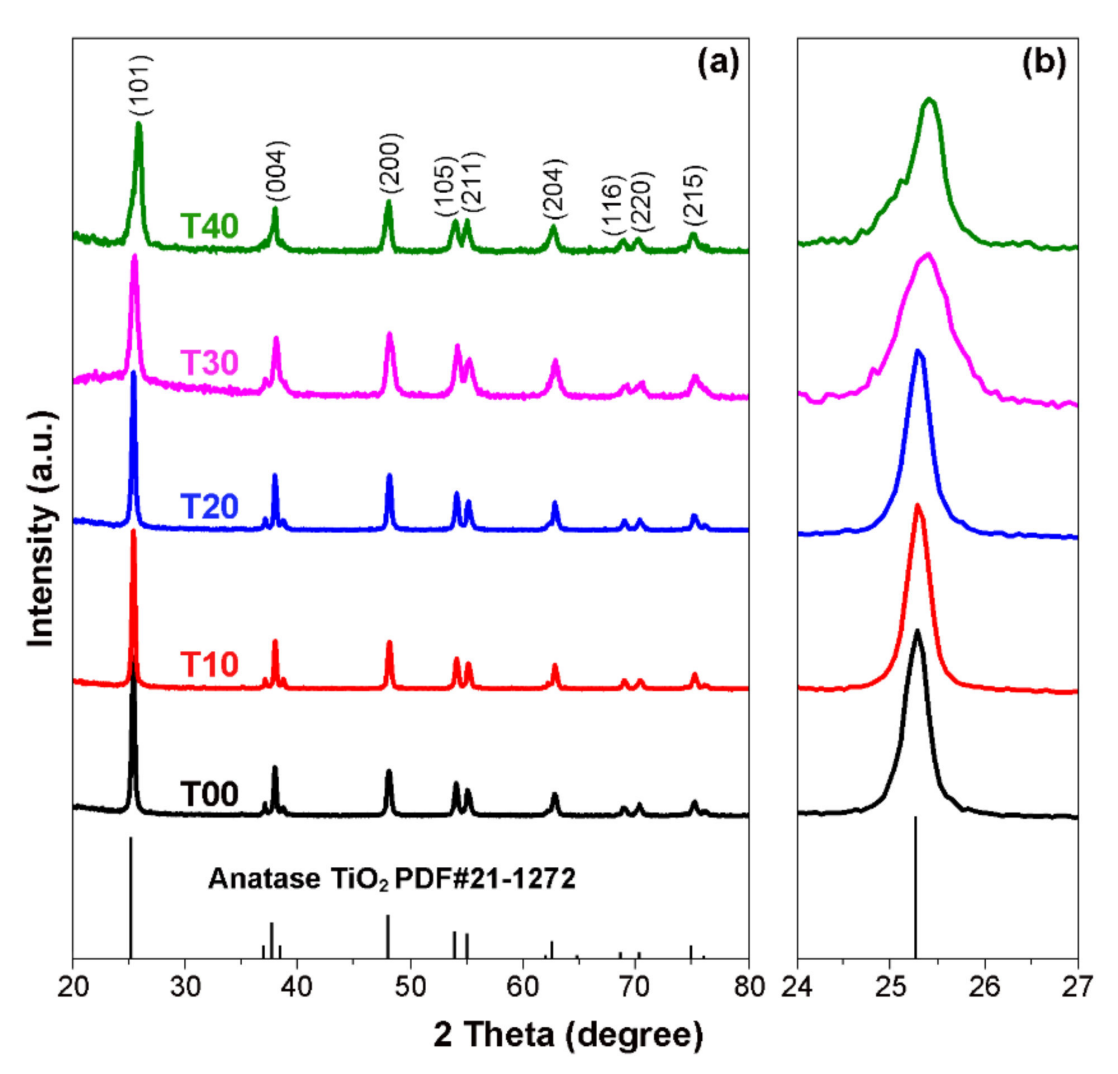
(**a**) XRD spectra of TiO_2_ photocatalysts obtained by solvothermal treatment of the sol at different triethanolamine concentrations and (**b**) enlarged XRD results.

Ti^3+^ self-doped TiO_2_ nanoparticles were prepared with different concentrations of TEA (0, 10, 20, 30, and 40 mL) via the solvothermal method at 180 °C for 20 h. The corresponding TiO_2_ nanoparticles were denoted as T00, T10, T20, T30, and T40, respectively. The morphology, structure, and composition of nanomaterials are known to have a direct impact on their PC performance. X-ray diffraction (XRD) was applied to study the structure and crystallization of obtained photocatalysts. As depicted in Figure 1a, those peaks located at 25.3°, 37.9°, 48.0°, 54.1°, 54.9°, 62.7°, and 68.9° represented the (101), (004), (200), (105), (211), (204), and (216) crystal planes of pure TiO_2_, maintaining the standard card of anatase TiO_2_ (JCPDS No. 21–1272) [16,54]. The peaks of TiH_2_ were not found in the XRD results, which proved that TiH_2_ was converted into anatase TiO_2_. Furthermore, with the increase in TEA content from 10 to 40 mL, the intensities of diffraction peaks decreased gradually, suggesting a decreased crystallinity for TiO_2_. It might be attributed to the formation of defects with high content of TEA (Figure 1b). With the increase in TEA amount, the half-width was gradually increasing, indicating that the crystallinity of the sample was deteriorated. The above XRD results show that the content of TEA had a direct effect on the crystallinity of TiO_2_ photocatalysts.

**Figure 2:**
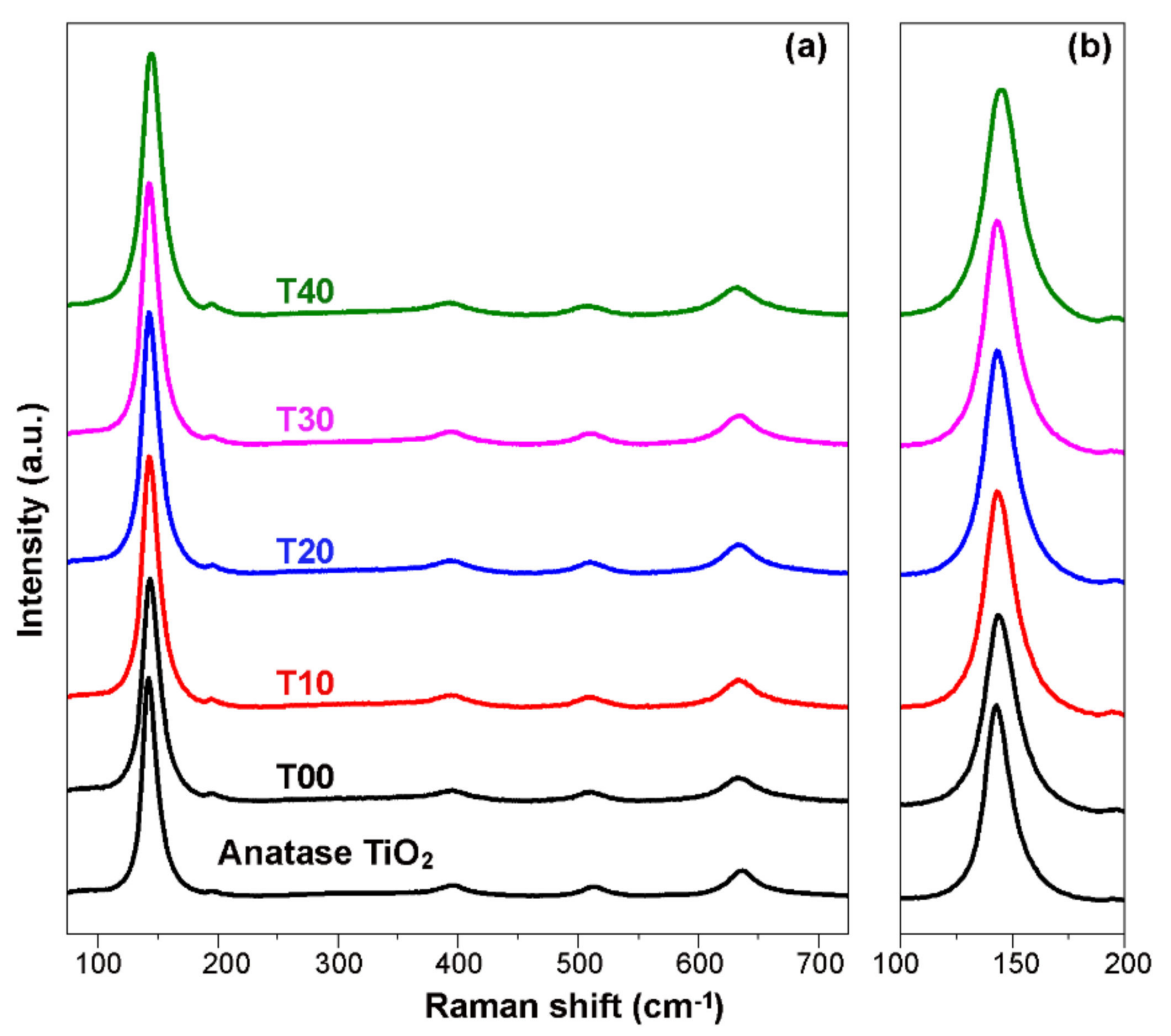
(**a**) Raman spectra of TiO_2_ photocatalysts obtained by hydrothermal treatment the sol at different triethanolamine concentrations and (**b**) enlarged Raman results.

As a simple, effective, and high-sensitivity detection technology, Raman spectra, originating from the vibration of molecular bonds, were employed to explore the significant structural changes in TiO_2_. As shown in Figure 2a, these peaks located at 145, 398, 514, and 636 cm^−1^ could index, respectively, to Eg, B1g, A1g, and Eg lattice vibration modes, indicating that all these nanomaterials were mainly anatase TiO_2_ [55]. As illustrated in Figure 2b, the strongest Raman bands around 145 cm^−1^ shifted to a lower wavenumber along with the peak broadening as the content of TEA increased from 10 to 40 mL, which might be attributed to the increase in the aspect ratio of spindle-shaped TiO_2_ nanoparticles. At the same time, the shift and broadening content reached the highest with 40 mL TEA, indicating that there existed a large number of defects in T40.

**Figure 3:**
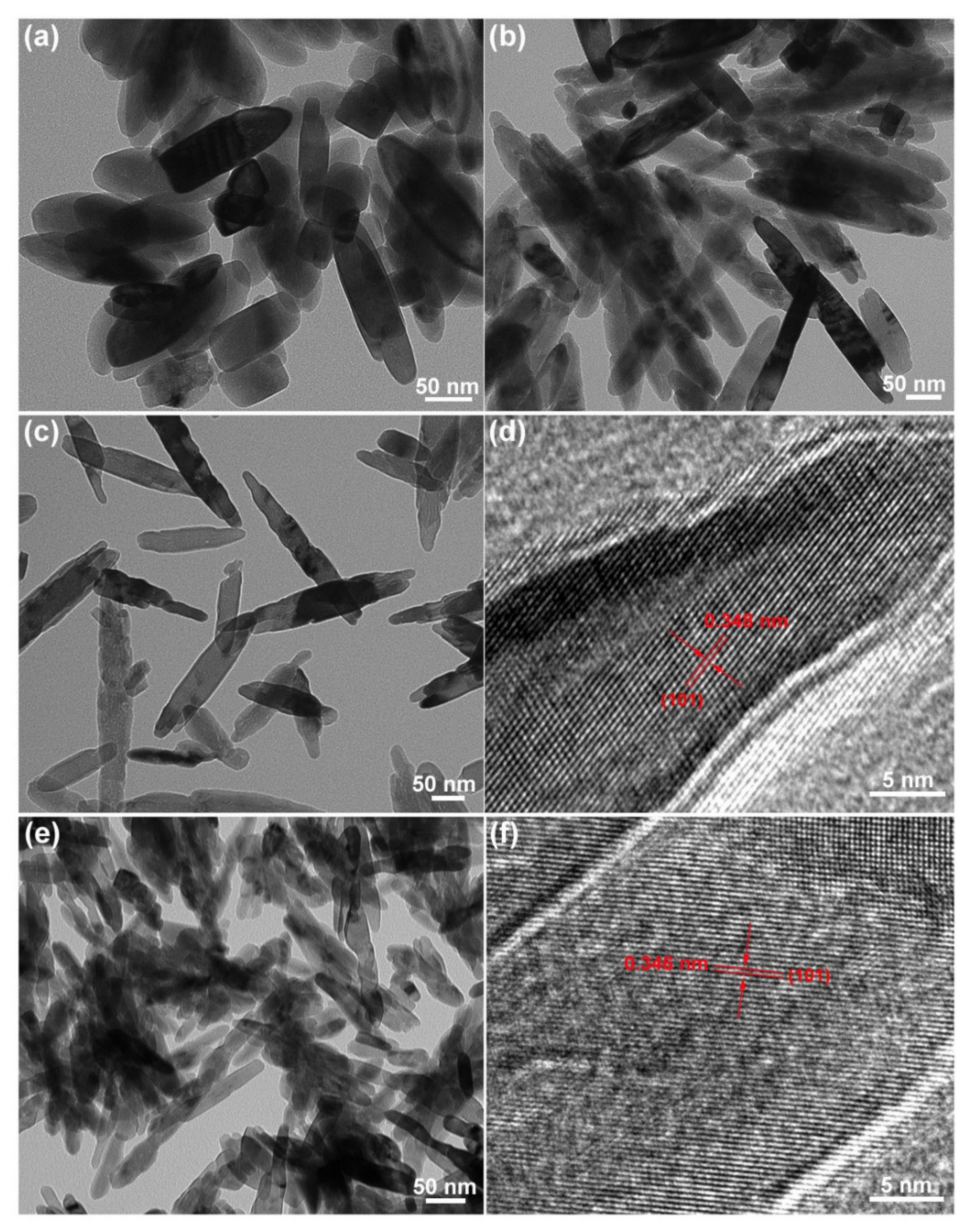
TEM and HRTEM pictures of TiO_2_ photocatalysts obtained with different content of TEA: (**a**) T10, (**b**) T20, (**c**,**d**) T30, and (**e**,**f**) T40.

Transmission electron microscopy (TEM) and HRTEM were employed to investigate the representative microstructures and crystalline properties of as-prepared nanomaterials. As shown in Figure 3, as-obtained samples were spindle-shaped particles. It was easy to find that the TEA concentration in the medium significantly affected the morphology of TiO_2_ nanomaterials. In absence of TEA, the obtained nanomaterials were a spherical-like particle (Figure S1a,b). The morphologies of as-obtained TiO_2_ nanomaterials become spindle-shaped in the presence of TEA. With the increase in TEA amount, the spindle-shaped nanorods became narrower and longer (Figure 3a–c). However, when the amount of TEA reached to 40 mL, the obtained sample become fragmented (Figure 3e), which further indicated that the amount of TEA had an important effect on the morphology of the TiO_2_ nanomaterials. The HRTEM results demonstrated that the crystallinity of the TiO_2_ photocatalysts decreased gradually with the increase in TEA (Figure 3d,f and Figure S1c,d). The crystal lattices of T00 were measured at 0.351 nm ), which matched the (101) crystal plane of anatase TiO_2_. While with increase in TEA, the crystal lattices of T10, T20, T30, and T40 decreased from 0.351 nm to 0.346 nm (Figure S1c,d and Figure 3d,f), indicating that the modification of TEA would affect the crystallinity of TiO_2_ nanomaterials.

**Figure 4:**
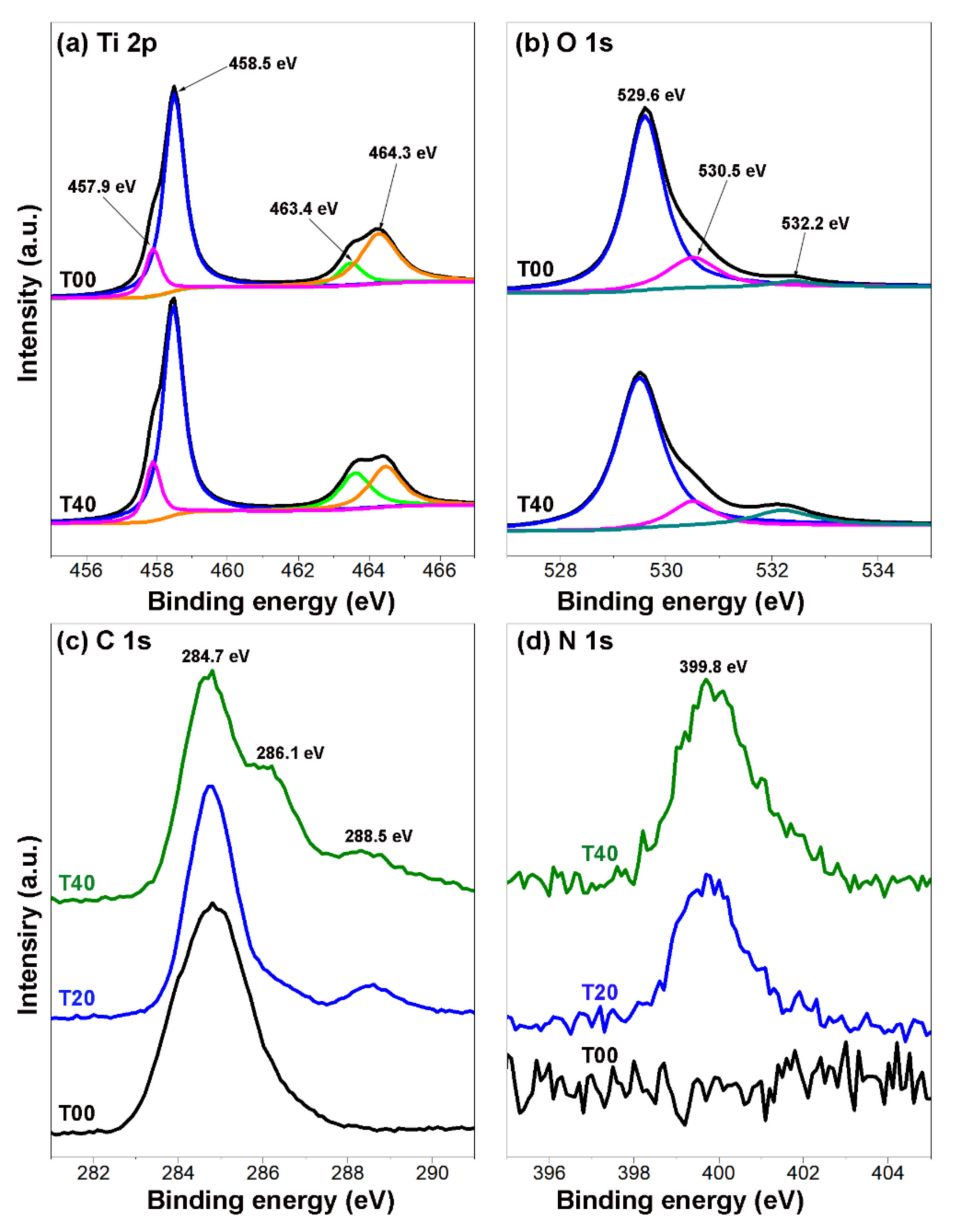
High-resolution XPS spectrum of different samples.

The XPS analysis is applied to further study the surface chemical environment of as-obtained TiO_2_ photocatalysts. The survey XPS patterns showed that as-fabricated TiO_2_ photocatalysts all contain O, C, and Ti (Figure S2). With the increase in TEA amounts in the medium, the N element binding energy peak appeared, indicating that the product contains N element on its surface. As illustrated in Figure 4a, the high-resolution XPS spectrum of Ti 2p for T00, T40 showed two peaks, which can be fitted into four peaks at 457.9, 458.6, 463.4, and 464.3 eV, assigning to Ti^3+^ 2p_3/2_, Ti^4+^ 2p_3/2_, Ti^3+^ 2p_1/2_, and Ti^4+^ 2p_1/2_, respectively [56,57]. Similarly, the XPS spectrum of O 1s for T00 and T40 also showed two peaks, which can be fitted into three peaks at 529.6, 530.5, and 532.2 eV, assigning to Ti–O bond, O–H, and OVs [58,59]. Compared with T00, the content of Ti^3+^ and OVs in the sample with TEA was greatly increased, indicating that the addition of TEA could not only form a one-dimensional spindle product but also regulate the content of Ti^3+^ and OVs in the product. Through comparative analysis of the binding energy peaks of Ti^3+^ 2p_3/2_ in all obtained samples, the content of Ti^3+^ was found to increase with the increase in TEA amount in the mixed solvent, which further demonstrated the influence of solvent effect on the product composition (Supporting Information, Figure S3). On one hand, a one-dimensional structure was conducive to the separation of photogenerated electrons and holes [55]. On the other hand, the introduction of Ti^3+^/OVs could lower the band-gap of TiO_2_, which facilitated the application of visible light and the improvement of PC performance. The C 1s spectra of T00, shown in Figure 4c, was deconvolved into one peak located at 284.7 eV, assigning to the C–C bond with sp^2^ orbital [60]. As comparison, C 1s spectra of T20 were fitted into two peaks at 284.7 and 288.5 eV, the latter of which was assigned as C–N bond. Similarly, the C 1s spectra of T40 were deconvolved into three peaks at 284.7, 286.1, and 288.5 eV, among which the peak at 286.1 eV was assigned to C–H with sp^3^ hybridization, indicating the introduction of TEA. Moreover, high-resolution N 1s spectra of T20 and T40 showed one more peak at 399.8 eV compared to the N 1s spectra of T00, which was assigned to the C–N–C bond (Figure 4d), further confirming the introduction of TEA on the surface of TiO_2_. A binding energy peak of Ti–C or Ti–N bond was not in all samples, which indicated that the C or N doping is not formed during the solvothermal treatment, and TEA only attached to the surface of TiO_2_ by physical adsorption. In addition, the FT-IR analysis results also proved that TEA anchored onto TiO_2_ photocatalysts (Supporting Information, Figure S4).

**Figure 5:**
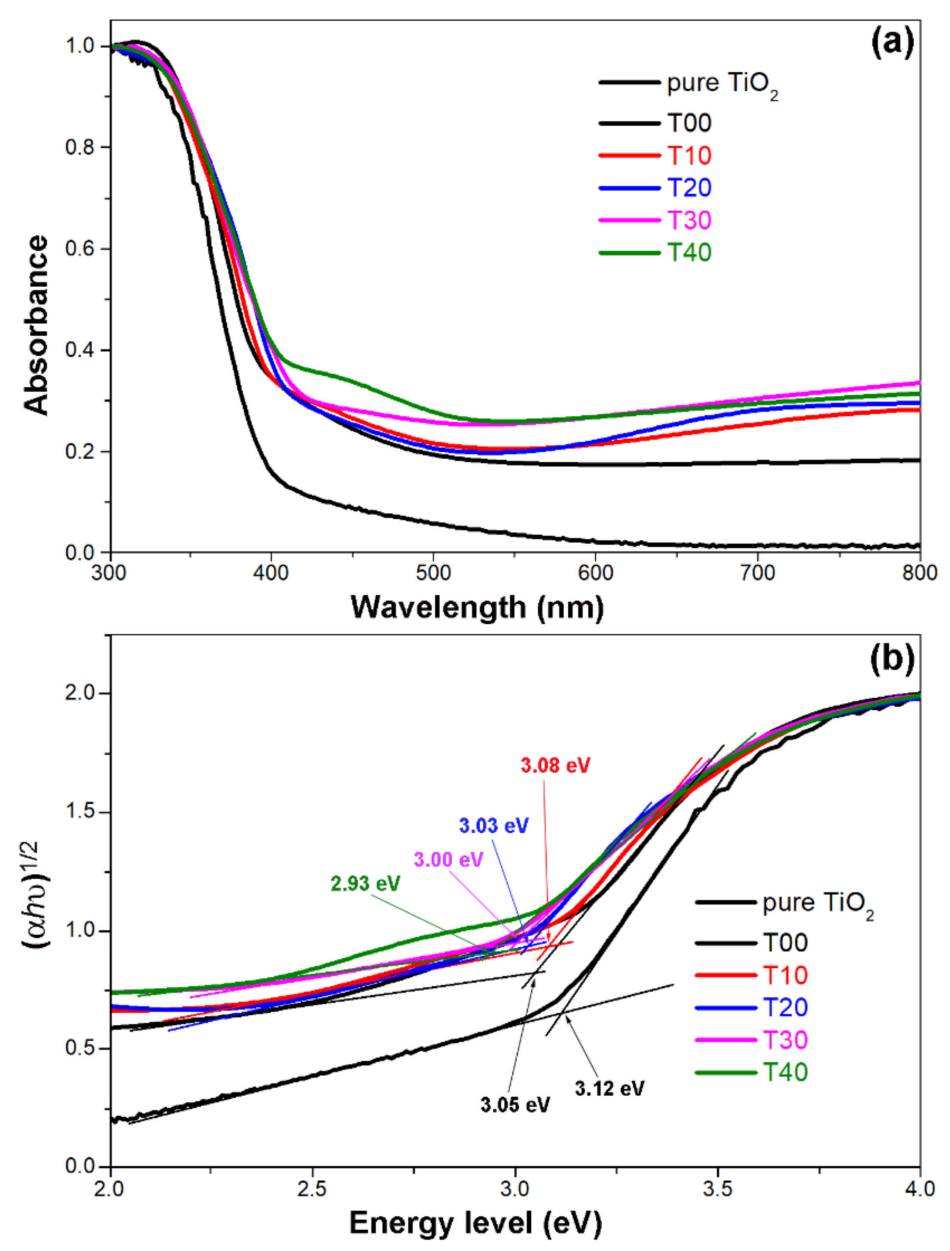
(**a**) UV-vis diffuse reflectance spectrum of TiO_2_ photocatalysts; (**b**) Plotting curves of Kubelka–Munk against photon energy over pure TiO_2_, T00, T10, T20, T30 and T40, respectively.

The optical performance of as-fabricated TiO_2_ photocatalysts was investigated by UV-vis diffuse reflectance spectra (DRS). As illustrated in Figure 5a, with TEA as a structure-directing and capping agent, the photo-response performance of TiO_2_ photocatalysts was greatly improved, extending to visible and infrared light regions. Furthermore, T40 exhibited the highest UV-vis absorption property than the other samples, indicating that the introduction Ti^3+^/OVs could produce a novel vacancy band located below the conduction band edge of anatase TiO_2_. As illustrated in Figure 5b, the band-gap energy of T40 was calculated to be 2.93 eV via plotting Kubelka–Munk formula against the photon energy. The band energy of T30, T20, T10, T00, and anatase TiO_2_ were calculated to be 3.00, 3.03, 3.08, 3.05, and 3.12 eV, which were larger than that of T40.

**Figure 6:**
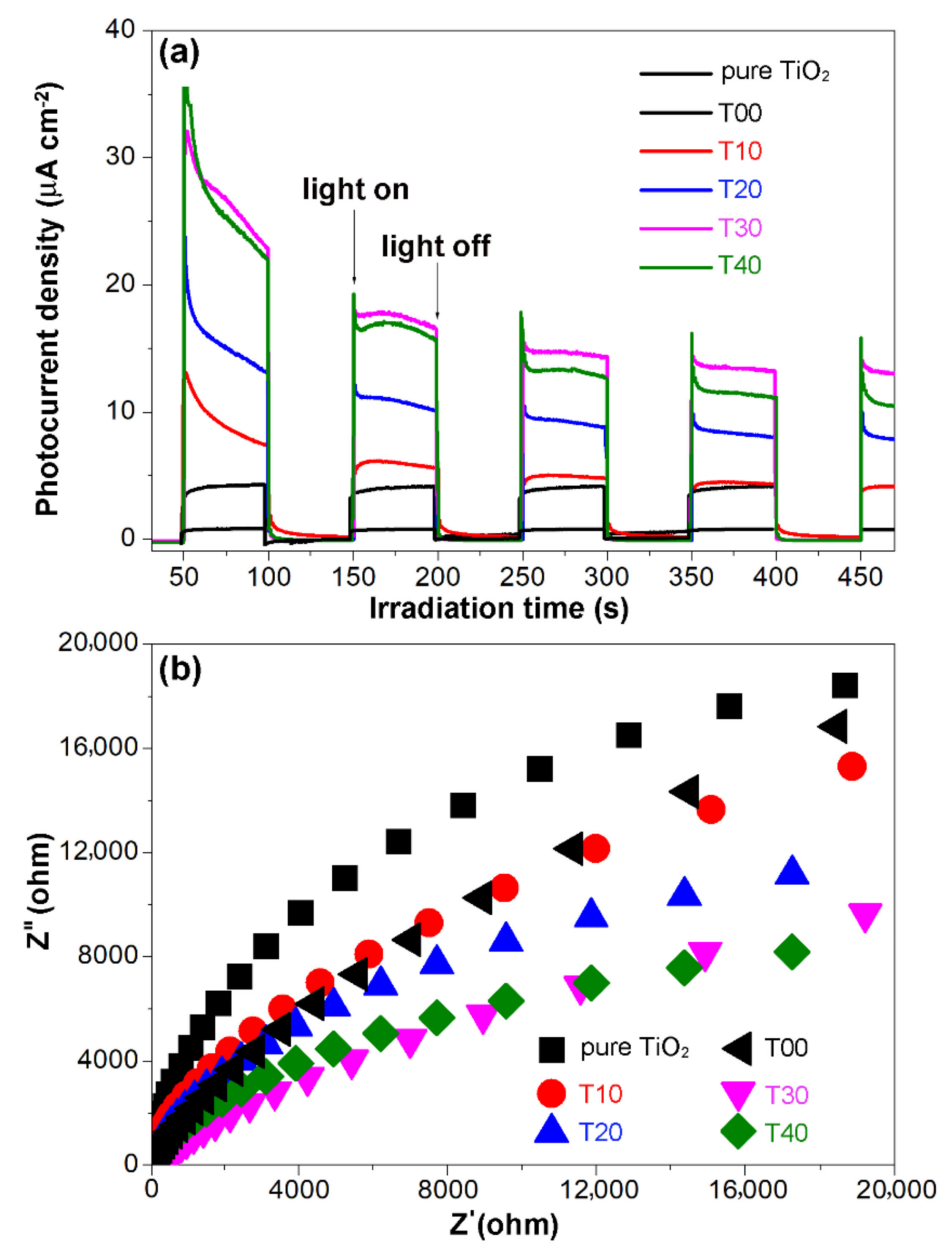
Transient photocurrent response (**a**) and EIS spectrum (**b**) of Nyquist plots acquired at open-circuit potential exposed to visible light illumination for different samples.

Photoelectrochemical transient photocurrent response under visible light can feasibly investigate the photogenerated electrons transfer property [61]. The instantaneous photocurrent data of those photocatalysts were investigated under visible light irradiation at an interval of 50 s. As displayed in Figure 6a, the photocurrent density of T10 was measured as 1.3 μA cm^−2^, while that of T20 and T30 were 2.8 and 3.6 μA cm^−2^, respectively. The photocurrent disappeared immediately after the visible light was removed, indicating that the photocurrent was completely generated by the photoelectrodes. Charge separation and transfer of photocatalysts were obtained by electrochemical impedance spectroscopy [62]. Figure 6b shows that the radius of the arch for T30 was smallest among those prepared TiO_2_ photocatalysts, indicating that T30 exhibited the lowest impedance. The above experimental results showed that Ti^3+^ self-doped and spindle structure were beneficial to the separation and transfer efficiency of photogenerated electron-holes.

**Figure 7:**
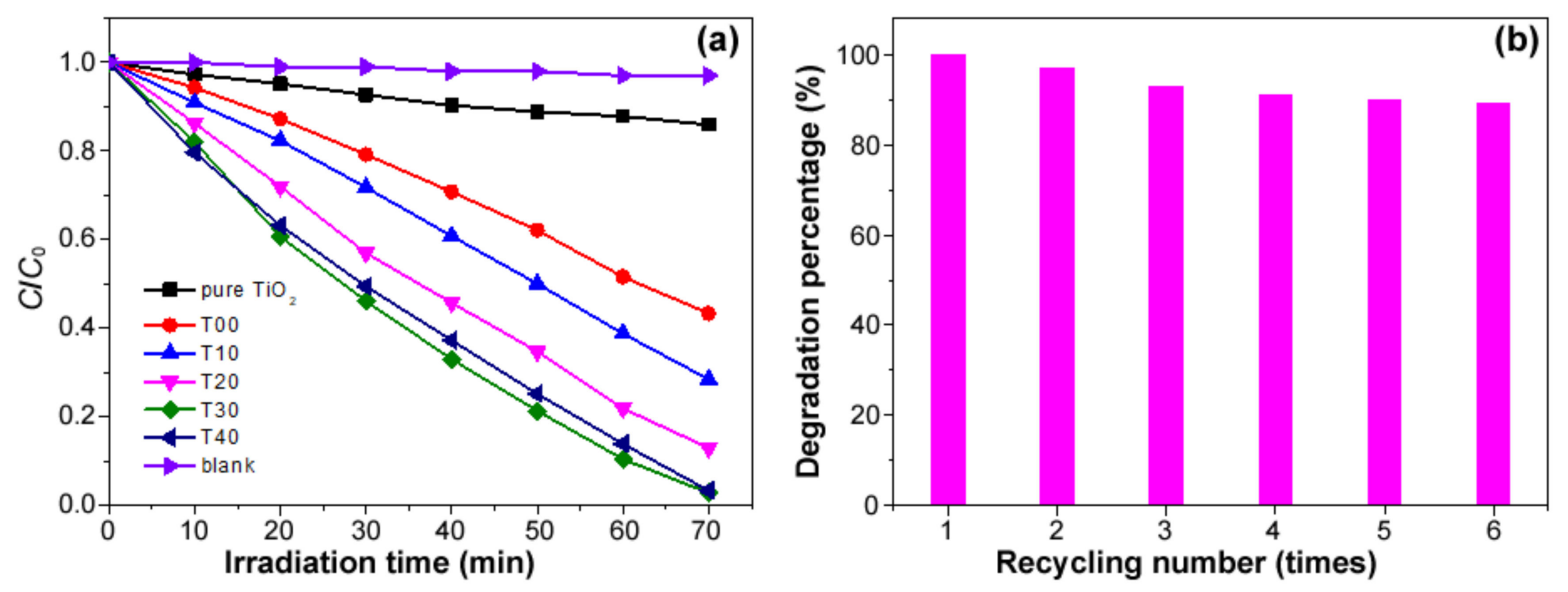
(**a**) Photocatalytic performance of the different samples; (**b**) recyclability of T30 in degradation of Rhodamine B.

The PC performance of as-prepared TiO_2_ photocatalysts was investigated by the degradation of Rhodamine B (RhB) when exposed to visible light. The time-dependent curve of concentration and spectrum during RhB degradation is shown in Figure 7a. Figure 7a also shows that the photodegradation of RhB dyes was not observed without photocatalyst. This indicates that RhB cannot be degraded under visible light while T30 sample showed the strongest PC activity, which was mainly attributed to its aspect ratio, allowing the generation of photogenerated electrons and holes and electron transfer to the surface. The stability of photocatalyst had an important influence on its practical application. Recycling experiment was employed to investigate the stability of as-prepared photocatalysts. As illustrated in Figure 7b, after six cycles, the PC performance of T30 had almost no loss, indicating that as-obtained possessed high PC stability and high practical application had potential.

**Figure 8:**
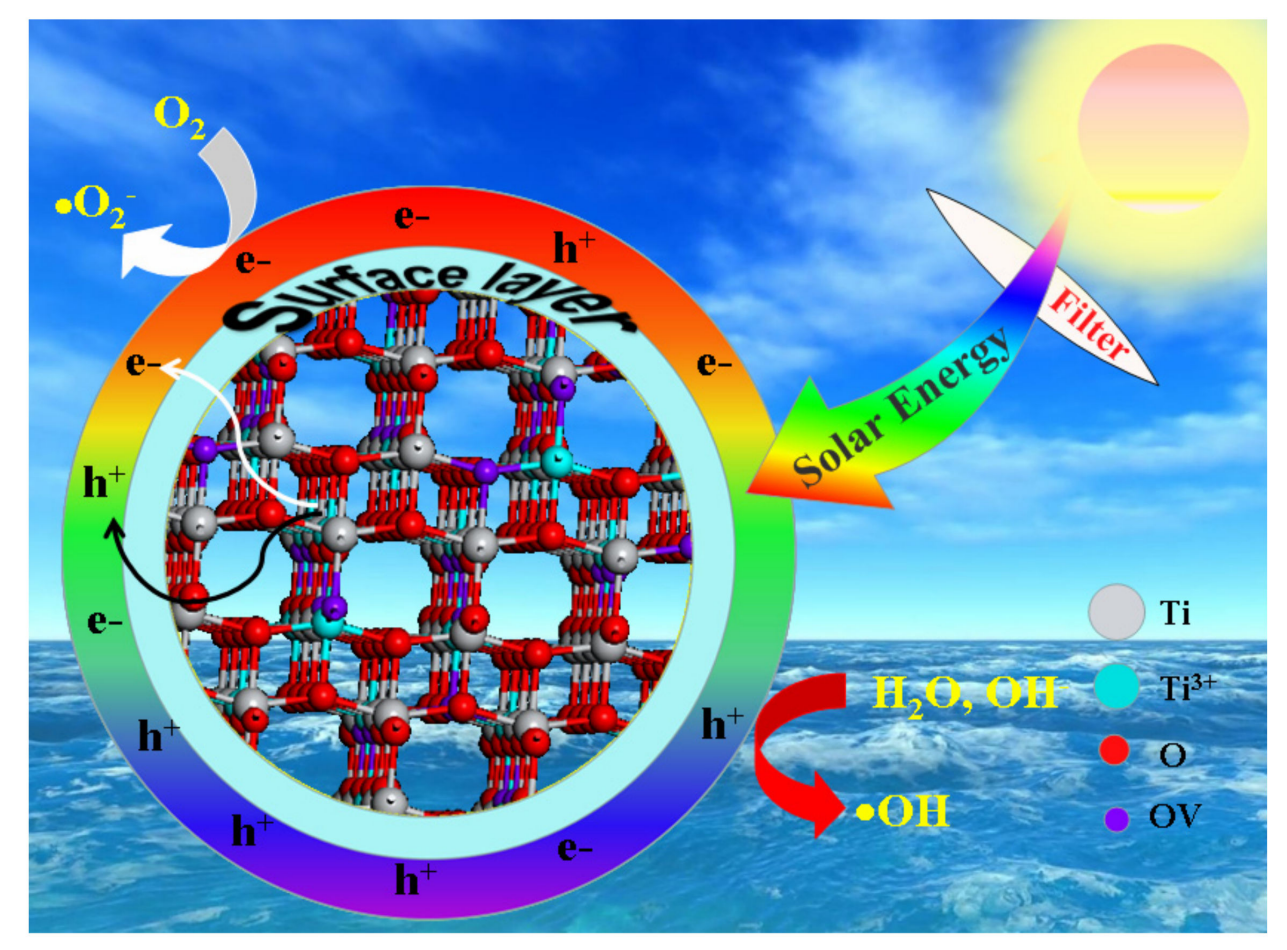
Schematic illustration of the visible-light photocatalytic mechanism of the Ti^3+^ self-doped TiO_2_ nanoparticles.

Generally, the PC activity is determined directly by the separation efficiency of photogenerated electrons and holes. In addition, it is widely recognized that photocatalysts with a larger specific surface can supply more surface active sites for the adsorption of RhB molecules, resulting in an enhanced PC performance. The BET surface results show that T00 sample has the largest surface area, while T30 sample has the smallest surface (Supporting Information, Figure S5); however, T30 has the best PC performance, which indicates that the specific surface areas are not the main factor affecting the PC performance. In addition, Figure 6 shows that T30 sample has the largest photocurrent and the smallest impedance, indicating that T30 has the higher separation effect of photogenerated electrons and holes. Therefore, the enhanced PC activity of Ti^3+^ self-doped TiO_2_ photocatalysts is attributed to the following reasons: (i) The introduction of Ti^3+^/OVs defects are used to reduce the band-gap of TiO_2_ into the visible light range and subsequently the new defect states appear below conduction band, and (ii) the spindle-shaped nanoparticles are beneficial to the separation of photogenerated electron holes and electron transport [55]. In addition, the introduction of Ti^3+^/OVs enhances the charge separation and diffusion while suppressing charge carrier recombination. When exposed to visible light, photogenerated electrons could change from valence band to conduction band, promoting the migration of electrons and holes to the surface TiO_2_ nanoparticles. As displayed in Figure 8, photogenerated electrons react with dissolved O_2_ to produce superoxide anion radicals (•O_2_^−^), while holes oxidize the surface OH^-^ groups and H_2_O molecules to generate •OH. The highly reductive •O_2_^−^ and oxidizing •OH will decompose RhB molecules into small micromolecules, such as H_2_O and CO_2_. Nevertheless, excessive Ti^3+^ would lead to the formation of the recombination center for photogenerated electron–hole pairs, which were harmful to the improvement of photocatalytic performance of TiO_2_.

## 4. Conclusions

In conclusion, spindle-shaped Ti^3+^ self-doped TiO_2_ photocatalysts were fabricated by a simple solvothermal strategy with triethanolamine as a structure-directing agent. The morphology and photochemical properties of TiO_2_ photocatalysts could be effectively controlled by adjusting the amount of trimethylamine. In addition, the crystallinity, photogenerated electrons, and holes of the TiO_2_ catalysts could also be effectively controlled by changing the amount of trimethylamine. As-prepared TiO_2_ photocatalysts were also employed for the photocatalytic degradation of rhodamine B. It was found that T30 exhibited the highest catalytic efficiency, which was mainly due to the large aspect ratio. Hence, the Ti^3+^ self-doped TiO_2_ photocatalysts with large aspect ratio will be a feasible alternative strategy for water treatment and organic degradation in the future.

## Data Availability

Data presented in this article are available at request from the corresponding author.

## Supplementary Materials

The following supporting information can be downloaded at: https://www.mdpi.com/article/10.3390/nano12132298/s1, Materials and Methods. Figure S1: TEM and HRTEM images of the samples obtained at different triethanolamine concentrations; Figure S2: Survey XPS spectra of the obtained samples at different TEA amount; Figure S3: High-resolution XPS spectrum of Ti^3+^ 2p3/2 for different samples; Figure S4: FTIP spectra for pure TiO_2_ and obtained samples with different TEA amount; Figure S5: N_2_ adsorption-desorption isotherms and the corresponding BET surface area of the different samples.
