# Supplementary material for: Construction of Spindle-Shaped Ti3+ Self-Doped TiO2 Photocatalysts Using Triethanolamine-Aqueous as the Medium and Its Photoelectrochemical Properties
Source: Nanomaterials (Basel). 2022 Jul 4;12(13):2298. doi: 10.3390/nano12132298 (PMC9268407; doi:10.3390/nano12132298)
Supplement: Supplementary file 1 [file nanomaterials-12-02298-s001.zip › nanomaterials-1783276-supplementary.pdf]

Supplementary Materials

Construction of Spindle-Shaped Ti^{3+} Self-Doped TiO_2 Photocatalysts Using Triethanolamine-Aqueous as the Medium and Its Photoelectrochemical Properties

Zunfu Hu [1], Qi Gong [1], Jiajia Wang [1], Xiuwen Zheng [2], Aihua Wang [3] and Shanmin Gao [2,3,*]

[1] School of Materials Science and Engineering, Linyi University, Linyi 276005, China; huzunfu@lyu.edu.cn (Z.H.); gongqi72909@163.com (Q.G.); wangjiajia@lyu.edu.cn (J.W.)
[2] School of Chemistry & Chemical Engineering, Linyi University, Linyi 276005, China; zhengxiuwen@lyu.edu.cn
[3] Yantai North China Microwave Technology CO., LTD., Yantai 264025, China; 13953598088@163.com
* Correspondence: gaoshanmin@lyu.edu.cn

1. Materials and Methods

1.1. Chemicals

Titanium hydride (TiH_2, 98%) was obtained from Sigma-Aldrich; triethanolamine hydrogen peroxide (H_2O_2, 30%) was obtained from Yuan Dong Chemical Co., Ltd.; polyvinylidene fluoride (PVDF) was bought from Cheng Du Chemical Co., Ltd.; N-methylpyrrolidone (NMP) was acquired from Da Mao Chemical Co., Ltd.

1.2. Preparation of Spindle-Shaped Ti^{3+} Self-Doped TiO_2 Photocatalysts

The spindle-shaped Ti^{3+} self-doped TiO_2 photocatalysts were synthetized as follows. First, 1.0 g TiH_2 powder, 5 mL H_2O, and 20 mL H_2O_2 were mixed in a beaker and underwent ultrasonication for 20 min. Then, H_2O_2 was added in batches until a yellow-green sol appeared, and the sol was divided into five portions. A total of 0, 10, 20, 30, and 40 mL of TEA and 40, 30, 20, 10, and 0 mL of water was added to the abovementioned sol, respectively (the samples denoted as T00, T10, T20, T30, and T40). The obtained mixture was stirred for 30 min and transferred into a Teflon-lined autoclave. The autoclaves were placed in a drying oven and annealed at 5 °C min^{-1} to 180 °C, kept for 20 h, and then cooled to room temperature at a cooling rate of 5 °C min^{-1}. After the reaction finished, the samples were centrifuged, washed by distilled water and ethanol three times, and then dried in an oven at 60 °C for 12 h.

1.3. Characterization

Rigaku D/max-2500VPC model X-ray diffractometer was used to characterize the crystalline phases of the final products by using Ni-filtered Cu-$K\alpha$ radiation at a scanning rate of 0.02° s^{-1}. TEM and HRTEM were performed on a JEOL-2100 microscope. The X-ray photoelectron spectrometer (Thermo Fisher ESCALAB Xi^{+}) with a monochromatic Al $K\alpha$ X-ray source (1486.6 eV photons) was used to characterize the chemical state of elements in the as-prepared samples. C 1s with 284.6 eV binding energy was selected as reference. The photocurrents were measured with an electrochemical analyzer (CHI 660E Chenhua Instrument Company). Fourier transform infrared (FT-IR) spectra were recorded on a MAGNA 550 FT infrared spectrometer on samples embedded into KBr pellets. Raman spectra were collected via a Jobin Yvon HR 800 micro-Raman spectrometer in the region of 100 cm^{-1} to 1000 cm^{-1} at 457.9 nm. The Brunauer–Emmett–Teller (BET) specific surface areas of the powders

were analyzed by nitrogen adsorption–desorption at 77 K in a Micromeritics ASAP 2020 nitrogen adsorption apparatus (USA).

1.4. Photocatalytic Experimental

Photocatalytic degradation of rhodamine-B was used to evaluate the photocatalytic activity of different samples. A total of 80 ml of RhB (10 mg/L) was weighed into a 100 ml jacketed beaker, and 0.04 g of catalyst was added, ultrasonically dispersed for 10 min, and magnetically stirred for 30 min in the dark. The mixture was then placed and irradiated under a 300 W Xe arc lamp (PLS-SXE300, Beijing Trusttech Co. Ltd, China) with UV cutoff filter (providing visible light $\lambda > 400$ nm). In order to retain the experiment temperature, the jacketed beaker was connected to the constant temperature water tank, and the water temperature was set to 25 °C. In order to prevent evaporation of water, the jacketed beaker had a quartz plate cover. The variation in RhB concentration was measured with a visible spectrophotometer by the absorbance throughout the PC process. During photoreactions, no oxygen was bubbled into the suspension.

1.5. Photoelectrochemical Measurement

The photoelectrochemical performance of the photocatalysts were tested on a Model 660E electrochemical workstation using a standard three-electrode system at room temperature. A self-made electrode was used as a working electrode; a platinum wire electrode and a calomel electrode were used as a counter electrode and a reference electrode, respectively. A total of 0.1 mol/L of Na_2SO_4 solution was used as an electrolyte to support electron transfer. The xenon light source is the same as above. The fluorine tin oxide (FTO) glass pieces were successfully cleaned ultrasonically with alcohol, isopropyl alcohol, and deionized water, and then dried in an air stream. A 0.02 g sample was obtained and then dispersed in 0.002 g of polyvinylidene fluoride in 1.0 ml N-methylpyrrolidone, ground in an agate mortar for 10 min. After that, we fabricated photo-anode by spin-painting the mixture suspension onto a FTO glass substrate. The coated area on the FTO glass was controlled to be 2×2 cm^2. Finally, the coated FTO glass was dried at 50 °C in vacuum to obtain the working electrode. The electrochemical impedance spectroscopy (EIS) measurements of the samples were performed in the dark or under illumination at open circuit potential over a frequency range of 10^4 to 1 Hz.

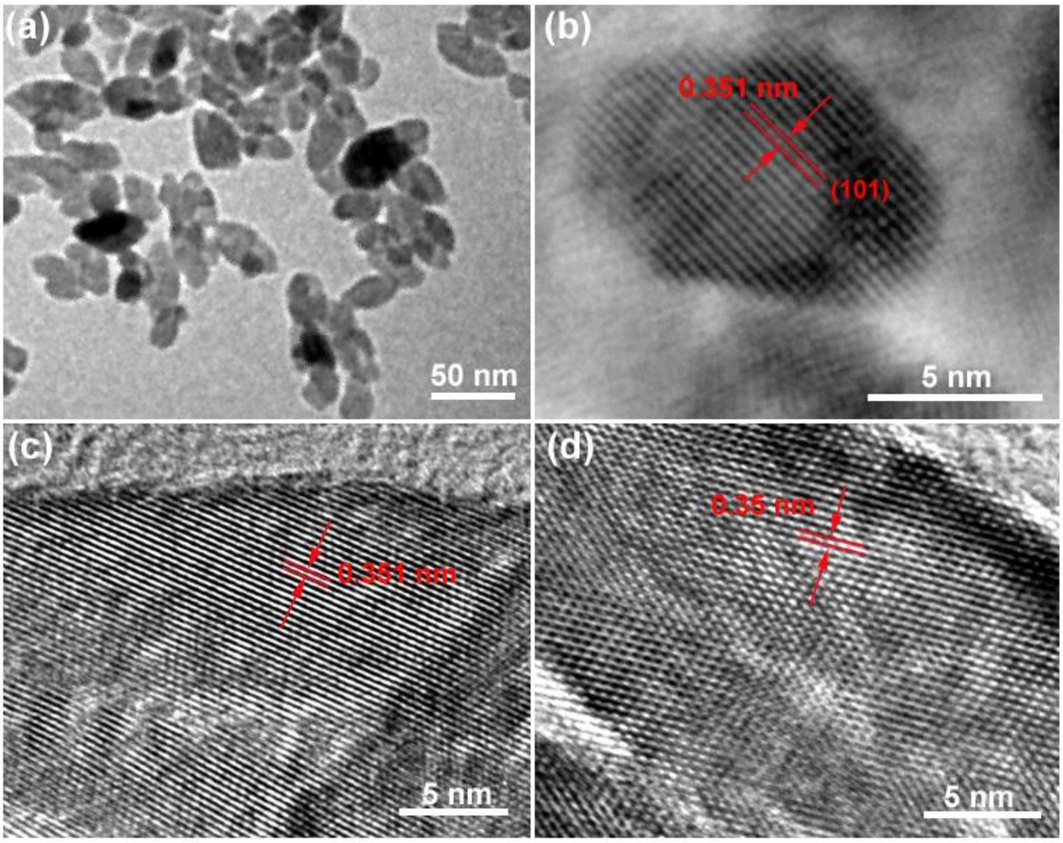

Figure S1. TEM and HRTEM images of the samples obtained at different triethanolamine concentrations. (**a**,**b**) T00, (**c**) T10, and (**d**) T20.

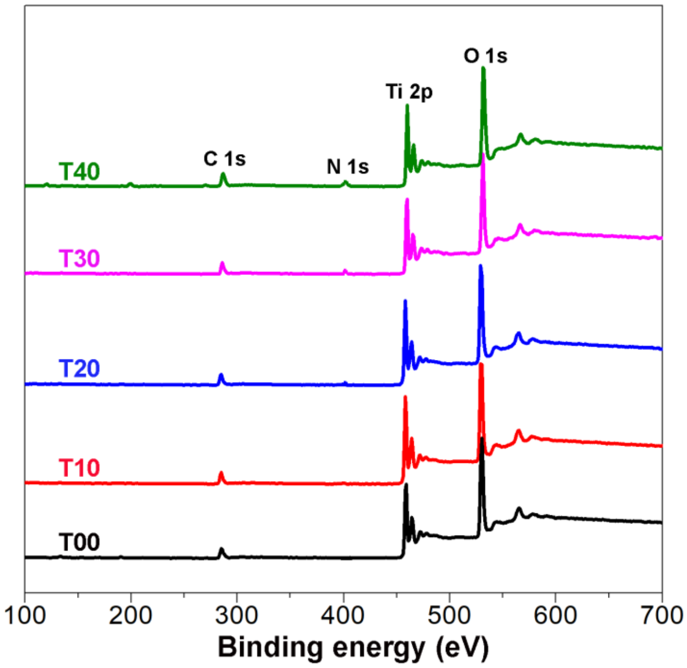

Figure S2. Survey XPS spectra of the obtained samples at different TEA amount.

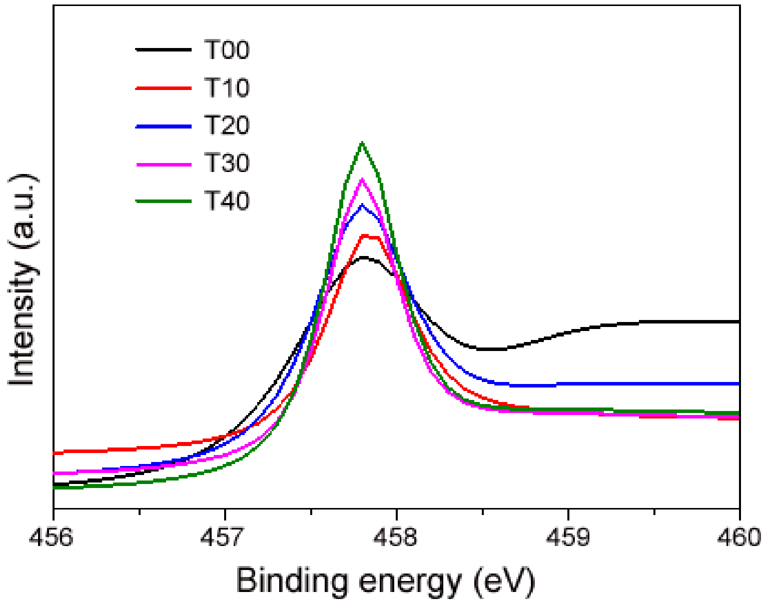

Figure S3. High-resolution XPS spectrum of Ti^{3+} $2p_{3/2}$ for different samples.

FT-IR was employed to study the surface functional groups of Ti^{3+} self-doped TiO_2 nanoparticles, as shown in Figure S4. For comparison, FT-IR result of pure TiO_2 nanoparticles was also given. The absorption peaks around 1636 and 3138 cm^{-1} were assigned to O–H stretching vibration and bending vibration of the absorbed H_2O. For Ti^{3+} self-doped TiO_2 photocatalysts with TEA as capping agent, those absorption peaks around 1068 and 1400 cm^{-1} were indexed to C–N–C and C–N stretching vibration. The C–N–C and C–N stretching vibration of T40 were found to be stronger than that of T00, indicating T40 contained more C–N and C–N–C bonds. With the increase in TEA, the intensities of these peaks enhanced, which might be attributed to the increase in C and N content on the surface. It also proved that TEA anchored onto TiO_2 photocatalysts. These results were consistent with the XPS results.

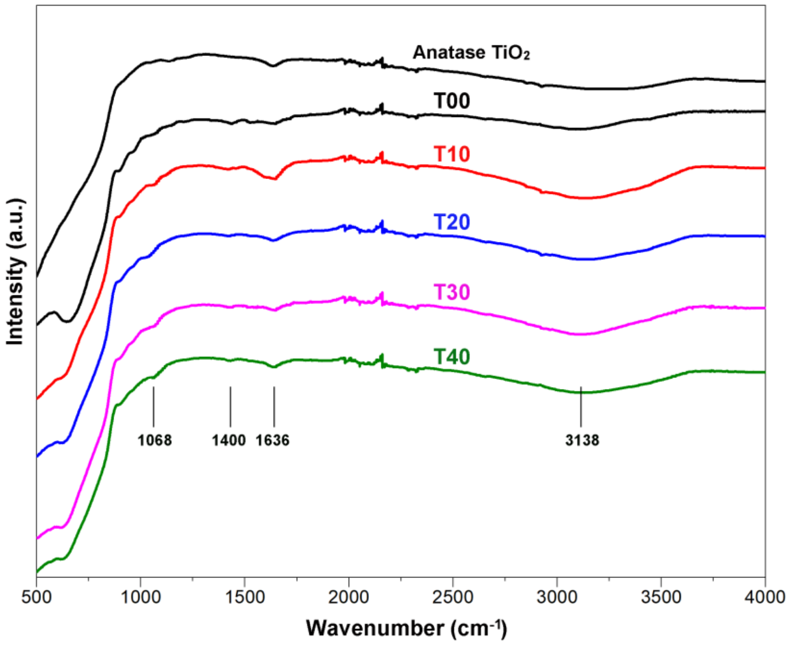

Figure S4. FTIR spectra for pure TiO_2 and obtained samples with different TEA amounts.

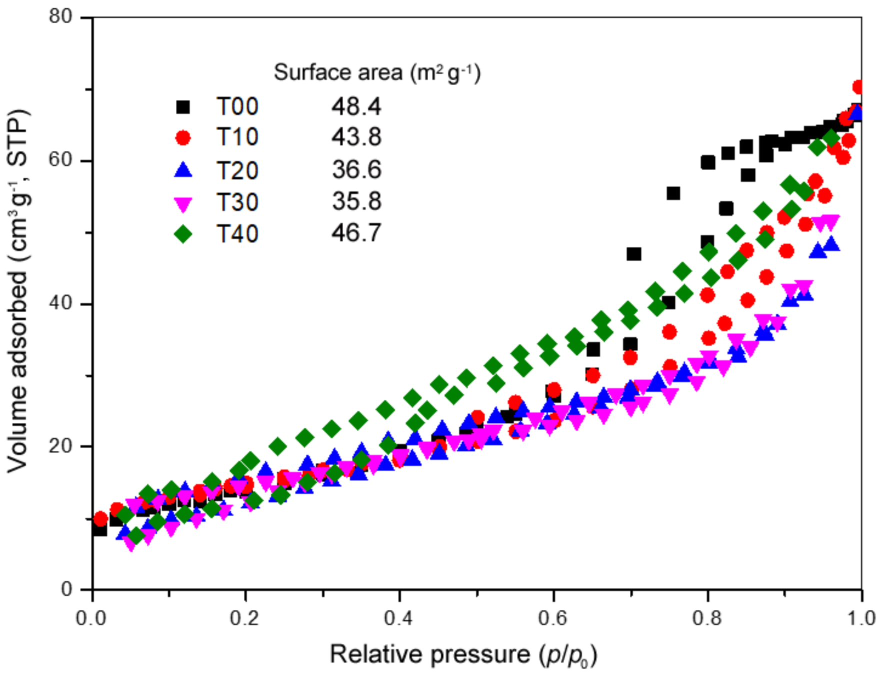

Figure S5. N_2 adsorption–desorption isotherms and the corresponding BET surface area of the different samples.

Nitrogen adsorption–desorption isotherms were recorded on the obtained samples to determine their surface areas. For measuring the BET specific surface areas, the samples were degassed at 150 °C prior to measurements, and the BET surface area was calculated from the linear part of the BET plot.
